# Estimation of dam line composition of 3-way crossbred animals using genomic information

**DOI:** 10.1186/s12711-022-00728-4

**Published:** 2022-06-15

**Authors:** Mario P. L. Calus, John M. Henshall, Rachel Hawken, Jérémie Vandenplas

**Affiliations:** 1 Animal Breeding and Genomics Centre, Wageningen University & Research, P.O. Box 338, 6700 AH Wageningen, The Netherlands; 2 Cobb-Vantress Inc., Siloam Springs, AR 72761-1030 USA

## Abstract

**Background:**

In genomic prediction including data of 3- or 4-way crossbred animals, line composition is usually fitted as a regression on expected line proportions, which are 0.5, 0.25 and 0.25, respectively, for 3-way crossbred animals. However, actual line proportions for the dam lines can vary between  ~ 0.1 and 0.4, and ignoring this variation may affect the genomic estimated breeding values of purebred selection candidates. Our aim was to validate a proposed gold standard to evaluate different approaches for estimating line proportions using simulated data, and to subsequently use this in actual 3-way crossbred broiler data to evaluate several other methods.

**Results:**

Analysis of simulated data confirmed that line proportions computed from assigned breed-origin-of-alleles (BOA) provide a very accurate gold standard, even if the parental lines are closely related. Alternative investigated methods were linear regression of genotypes on line-specific allele frequencies, maximum likelihood estimation using the program ADMIXTURE, and the genomic relationship of crossbred animals with their maternal grandparents. The results from the simulated data showed that the genomic relationship with the maternal grandparent was most accurate, and least affected by closer relationships between the dam lines. Linear regression and ADMIXTURE performed similarly for unrelated lines, but their accuracy dropped considerably when the dam lines were more closely related. In almost all cases, estimates improved after adjusting them to ensure that the sum of dam line contributions within animals was equal to 0.5, and within dam line and across animals the average was equal to 0.25. Results from the broiler data were much more similar between methods. In both cases, stringent linkage disequilibrium pruning of genotype data led to a relatively low accuracy of predicted line proportions, due to the loss of too many single nucleotide polymorphisms.

**Conclusions:**

With relatively unrelated parental lines as typical in crosses in pigs and poultry, linear regression of crossbred genotypes on line-specific allele frequencies and ADMIXTURE are very competitive methods. Thus, linear regression may be the method of choice, as it does not require genotypes of grandparents, is computationally very efficient, and easily implemented and adapted for considering the specific nature of the crossbred animals analysed.

**Supplementary Information:**

The online version contains supplementary material available at 10.1186/s12711-022-00728-4.

## Background

The implementation of genomic selection in pig and poultry breeding programs has renewed the interest to use crossbred information to estimate breeding values of purebred selection candidates for crossbred performance [1]. Depending on the type of crossbreeding, there may be variation in the line composition of crossbred animals. While F1 crossbred animals have exactly 50% of their alleles obtained from the sire and dam line, for 3-way crossbred animals the contribution of the dam lines varies around the expected value of 0.25. Likewise, for 4-way crossbred animals this variation is present for all four lines involved. In breeding value estimation models, fixed effects for each line composition type can be modelled as a regression on the expected proportions for each of the lines involved in the crossbreeding scheme, as a class effect when using a univariate model, or as separate mean effects in a multivariate model that specifies separate traits for different purebred and crossbred categories of animals [2, 3]. In all cases, it is typically assumed that animals belonging to a particular type of cross have the same line composition.

When the crossbred animals included in the breeding value estimation are genotyped, then their genotypes can be used to estimate their actual line proportions, which in turn can be included in the model instead of the expected line proportions. Actual line proportions can be estimated using efficient maximum-likelihood based methods such as ADMIXTURE [4], or linear regression of allele counts of an animal on frequencies of the corresponding allele in different breeds [5, 6]. Alternatively, methods can be used that identify different haplotypes in crossbred animals, and then estimate for each haplotype from which ancestral breed or line it was inherited. Subsequently, the estimated breed-origin-of-alleles (BOA) can be assigned for all alleles within a haplotype, and line proportions can be derived as the proportion of alleles assigned to a particular line [7]. By tracing the inheritance of long-range haplotypes from purebred to crossbred animals, BOA aims at avoiding confusion between breeds due to common short-range linkage disequilibrium (LD). Thus, line proportions that are derived from BOA arguably reflect identical-by-descent relationships to the purebred ancestor of a particular line. In contrast, linear regression and ADMIXTURE directly estimate the line proportions of crossbred animals as an average across the genome, and as such, at best, reflect the identical-by-state relationships to the purebred ancestor of a particular line. The more sophisticated modelling through BOA is expected to yield more accurate estimated line compositions than the other methods, especially for closely-related populations that may still share long haplotypes. Of all these methods, the linear regression approach is the easiest to implement, and likely also the most computationally efficient [8].

Empirical validation of methods to estimate line proportions in real data is challenging in the sense that a gold standard is needed to evaluate the estimated line proportions. In unstructured crosses, expected line proportions that can be computed from pedigree data vary across animals, and as such can be used to evaluate the line proportions that are estimated from genomic data [6, 9, 10]. This allows to verify whether the estimated line proportions are unbiased, both in terms of average level and dispersion of the estimates, but not whether they estimate the actual line proportions accurately. In addition, in structured 3- and 4-way crosses, expected values of animals in the same cross are all the same, and can only be used to evaluate average estimates within a cross. Therefore, there is a need to identify a method that can be used in applications to real data to generate results that can be considered as a gold standard, enabling to validate other methods that may be easier to implement and are computationally cheap.

Thus, our objective was to compare the performance of five methods in the estimation of dam line composition in 3-way crossbred animals using different methods. First, we validate the use of line composition computed from estimated BOA as a gold standard, using simulated data of 3-way crossbred pigs. Second, we develop two approaches: one based on the genomic relationship with the corresponding grandparent, and a linear regression approach that takes advantage of the expectations of the contributions of the different lines. Finally, the performance of these newly developed approaches is compared to the performance of ADMIXTURE, both on simulated pig data, and on real data of 3-way crossbred broilers.

## Methods

To evaluate the various methods to estimate line proportions for the dam lines (B and C) in 3-way crossbred animals [A(BC)], both simulated pig and actual 3-way broiler data were used. In this section, we start by describing the methods used, how they are compared to each other, and finally the datasets used.

### Methods to estimate dam line composition

Five different methods were considered to estimate the dam line composition of 3-way crossbred animals. The first method used is based on the estimated breed-origin-of-alleles, and hereafter referred to as BOA. The BOA method is described in detail by Vandenplas et al. [7], and involves three steps: (1) phasing genotype data of both purebred and crossbred animals simultaneously, (2) assigning the breed-of-origin to each of the haplotypes of the crossbred animals, and (3) finally assigning breed-of-origin to all single nucleotide polymorphism (SNP) alleles of the crossbred animals, based on the assigned breed-of-origin of the haplotype in which they reside. The computed line proportions obtained from the BOA results, are then computed as the proportion of SNP alleles coming from lines B and C. Note that this implicitly assumes that the SNPs used, are equally spaced throughout the genome. Small deviations from this assumption are expected to hardly affect the results when the number of SNPs used is sufficiently large.

The second method used the ADMIXTURE software [4, 11] in its “supervised” mode. Because ADMIXTURE assumes linkage equilibrium between the SNPs, it is recommended to prune the genotype data based on LD [4]. To test the sensitivity of the results to pruning of the data, we evaluated estimated line proportions using the full datasets against those obtained after pruning for r^2^ thresholds of 0.1, 0.3, 0.5, 0.7 or 0.9, respectively. Pruning was performed against an r^2^ threshold of 0.1, within a sliding window of 50 SNPs that was shifted by 10 SNPs each time, using the statement “--indep-pairwise 50 10 0.1” in PLINK [12].

The third method, hereafter referred to as LR, involves a linear regression of all SNP allele counts (coded as 0, 1, and 2) of each crossbred animal on the line-specific frequencies of the corresponding alleles computed in each of the purebred lines involved in the cross [6, 8]:

$${\mathbf{g}}_{\mathbf{i}}={\mathbf{x}}_{\mathbf{A}}{\mathrm{b}}_{\mathrm{A},\mathrm{i}}+{\mathbf{x}}_{\mathbf{B}}{\mathrm{b}}_{\mathrm{B},\mathrm{i}}+{\mathbf{x}}_{\mathbf{C}}{\mathrm{b}}_{\mathrm{C},\mathrm{i}}+{\mathbf{e}}_{\mathbf{i}},$$

where ${\mathbf{g}}_{\mathbf{i}}$ is a vector of SNP allele counts of crossbred animal $i$, ${\mathbf{x}}_{\mathbf{A}}$, ${\mathbf{x}}_{\mathbf{B}}$ and ${\mathbf{x}}_{\mathbf{C}}$ are vectors of the frequencies of the corresponding allele computed in purebred line A, B, and C animals, ${\mathrm{b}}_{\mathrm{A},\mathrm{i}}$, ${\mathrm{b}}_{\mathrm{B},\mathrm{i}}$ and ${\mathrm{b}}_{\mathrm{C},\mathrm{i}}$ are regression coefficients corresponding to the line proportions for lines A, B and C, and ${\mathbf{e}}_{\mathbf{i}}$ is a vector of error terms. Since we know that the line A proportion is 0.5 for all A(BC) animals, we can fill in this value for ${\mathrm{b}}_{\mathrm{A},\mathrm{i}}$ and adjust ${\mathbf{g}}_{\mathbf{i}}$ as follows: ${\mathbf{g}}_{\mathbf{i}}^{\boldsymbol{*}}={\mathbf{g}}_{\mathbf{i}}-{\mathbf{x}}_{\mathbf{A}} {*} 0.5$. The adjusted regression equation to compute line B and C proportions, then becomes:

$${\mathbf{g}}_{\mathbf{i}}^{\boldsymbol{*}}={\mathbf{x}}_{\mathbf{B}}{\mathrm{b}}_{\mathrm{B},\mathrm{i}}+{\mathbf{x}}_{\mathbf{C}}{\mathrm{b}}_{\mathrm{C},\mathrm{i}}+{\mathbf{e}}_{\mathbf{i}}.$$

The fourth method, hereafter referred to as REL_GP, takes advantage of the fact that in an A(BC) crossbred animal, all line B alleles originate from the line B maternal grandsire, and all line C alleles originate from the line C maternal granddam. Thus, the realized line proportions are equivalent to the proportion of their genome that they have inherited from their grandparents. Consequently, the line B (C) proportion is equal to the genomic identity-by-descent relationship between an A(BC) crossbred animal $i$ and its maternal grandsire (granddam) $j$, if this grandparent is not inbred. Therefore, as a proxy, we used the identity-by-state relationships between A(BC) crossbreds and their maternal grandparents (${\mathrm{G}}_{ij}$), which were computed as multi-population genomic relationships as described by Wientjes et al. [13] treating the purebred lines and the A(BC) crossbred animals as different populations. The required allele frequencies for the three purebred lines were obtained by regressing all available genotypes on the expected line proportions for the three lines involved. The required allele frequencies for the A(BC) crossbred animals were then obtained as a weighted average of the line-specific allele frequencies, using their expected line proportions, i.e., 0.5, 0.25 and 0.25, as weights.

The fifth method is similar to the fourth one, but avoids making the assumption that the grandparent is not inbred. The expected relationship of a 3-way crossbred animal with e.g., its line B grandsire is $0.25(1+{F}_{mgp}$), where ${F}_{mgp}$ is the inbreeding coefficient of the maternal grandparent, while the expected line B proportion of the 3-way crossbred animal is 0.25, regardless of the value of ${F}_{mgp}$. Thus, the fifth method, hereafter referred to as REL_GP_noF, is based on the relationship of crossbred animal $i$ with its grandparent $j$ divided by $(1+{F}_{mgp}$). Since the diagonal element of the maternal grandparent in the genomic relationship matrix $\mathbf{G}$ is an estimator for $1+{F}_{mgp}$, the fifth method estimated the line proportion as the adjusted relationship (${G}_{ ij}^{*}$) between animal $i$ and maternal grandparent $j$, computed as ${G}_{ ij}^{*}={\mathrm{G}}_{ij}/{\mathrm{G}}_{jj}$.

As mentioned previously, the expected line proportions for the dam lines in a 3-way cross are 0.25, the line proportion of the sire line is exactly 0.5, and the sum of the line proportions of the two dam lines is also exactly 0.5. Considering this, for all methods, estimated line proportions for the dam lines were post-processed partly following the procedure outlined by He et al. [14]. Any line proportions below the minimum possible value 0 were set to 0, and values above the maximum possible value 0.5 were set to 0.5. Thereafter, the mean estimated line B proportion was set equal to the expected value of 0.25 by adding a value of $0.25-{\overline{brfr} }_{A(BC)}^{B}$ to the estimated line B proportion for all A(BC) animals, where ${\overline{brfr} }_{A(BC)}^{B}$ is the average line B proportion across all A(BC) animals. The same was done for line C proportions. After adjustment of this mean, again line proportions below 0 or above 0.5, were set to 0 and 0.5, respectively. Finally, for each A(BC) animal, its line B and C proportions were linearly rescaled such that after rescaling their sum was equal to 0.5, i.e., for each animal ${\widehat{brfr}}_{A(BC)}^{B}=0.5-{\widehat{brfr}}_{A(BC)}^{C}$. As a result, estimated parameters such as the accuracy as explained hereafter, were identical for both dam lines by construction, and therefore are only presented for line B. Another consequence of this post-processing of the estimates, is that all five methods had the same mean estimated values, including those derived from the BOA, and the true line proportions.

### Evaluation of estimated dam line composition

For the simulated data, true line proportions were computed as the proportion of SNP alleles that an individual received from a particular line. The accuracy of the estimated dam line composition was computed as the correlation between the estimated and true line proportions. Dispersion bias of the estimated dam line composition was computed as the coefficient of the regression of true on estimated line proportions, with a value of 1 indicating no dispersion bias. To evaluate the estimation errors of the different methods, we also reported the maximum absolute error, and the root mean squared error (RMSE). Based on the simulated data, we evaluated whether line proportions that are estimated from BOA can be used as a semi-gold standard in practical data. Subsequently, BOA was used as a gold standard in the broiler data instead of the true line proportions, since these are not known.

Our hypothesis is that line proportions derived from BOA provide an appropriate gold standard to evaluate line proportions obtained with other methods. In addition to testing this hypothesis in the simulated data, we also evaluated whether or not the observed distribution of line proportions derived from BOA in the broiler data, was in line with the expected distribution based on theory. This expected distribution was assumed to have a mean of 0.25. The variance was computed using a formula that predicts the expected variance of identity-by-descent sharing between grandparent and grand-offspring pairs [15]. In this computation, we assumed that the grandparent is not inbred, following the same reasoning as that used to derive the method REL_GP_noF. The formula requires the number of chromosomes in the species considered, and the individual length of each chromosome in centiMorgan. For the simulated data, this information was obtained from Vandenplas et al. [7] as explained hereafter. For the broiler data, those required details were obtained from Groenen et al. [16], using the length of each chromosome averaged across the male and female linkage maps.

**Table 1:** Different quality measures of the estimated line B proportions derived with ADMIXTURE with various levels of pruning based on linkage disequilibrium (r^2^) in the simulated data^a^

Measure	Lines	r^2^ threshold for pruning					
		0.1	0.3	0.5	0.7	0.9	No.
Number of SNPs	Close	305	1364	2661	3781	4679	5720
	Distant	281	1190	2425	3543	4499	5753
	Unrelated	260	936	1953	3016	3989	5740
Accuracy	Close	0.560	0.694	0.696	0.689	0.682	0.675
	Distant	0.787	0.890	0.901	0.897	0.890	0.886
	Unrelated	0.891	0.958	0.966	0.965	0.963	0.959
Dispersion bias	Close	0.404	0.533	0.536	0.531	0.524	0.517
	Distant	0.653	0.808	0.824	0.820	0.809	0.801
	Unrelated	0.810	0.916	0.930	0.930	0.925	0.922
Maximum error	Close	0.404	0.330	0.332	0.334	0.341	0.348
	Distant	0.285	0.192	0.181	0.181	0.186	0.190
	Unrelated	0.181	0.110	0.104	0.103	0.109	0.112
RMSE	Close	0.132	0.106	0.106	0.107	0.108	0.109
	Distant	0.083	0.056	0.053	0.054	0.056	0.058
	Unrelated	0.056	0.034	0.031	0.031	0.032	0.034

*RMSE* root mean squared error

^a^All 428 A(BC) crossbreds were used

### Simulated data

The simulated data were generated and described in detail by Vandenplas et al. [7]. Three different scenarios were simulated. The three purebred lines A, B, and C, were separated for 5, 20 or 50 generations of random selection, to represent closely-related, distantly-related, and unrelated lines, respectively. For each of the scenarios, 10 replicates were simulated. The generated data in each scenario included SNP allele counts of  ~ 1000 purebred animals of each of the lines A, B, and C, and 428 A(BC) 3-way crossbred animals, of which on average 188 had both maternal grandparents genotyped. Alleles were generated for two chromosomes, with on average, across replicates and scenarios, 4800 segregating SNPs on the first chromosome of 3.20 Morgan, and 920 segregating SNPs on the second chromosome of 0.61 Morgan. These two chromosomes resembled the two pig *Sus scrofa* chromosomes (SSC), SSC1 and SSC18, respectively. The SNP density was comparable to that of a 60k SNP chip [17]. In our analyses, we only used SNPs that had a minor allele frequency (MAF) in the data across all line compositions higher than 0.1, to be consistent with the previous BOA analysis of this data [7], from which we used the results in our current study.

The simulated data did not include the allele counts of all generated animals, and as a consequence, not all maternal grandparents of genotyped A(BC) animals were included in the data. Estimated line compositions were evaluated only for crossbred animals that had the genotypes of both their maternal grandparents included in the data, since two of the used methods relied on the genotypes of the maternal grandparents. Across scenarios and replicates, this was on average 188 A(BC) animals. All 428 A(BC) crossbred animals were used only for the initial evaluation of ADMIXTURE with different levels of LD pruning.

### Broiler data

The broiler data was described in detail by Calus et al. [18], and that study also generated the BOA results used here. The data used here included allele counts for 55,729 segregating SNPs for purebred animals of each of the lines A (n = 8205), B (n = 372), and C (n = 720), and 10,943 A(BC) 3-way crossbred animals. In total, 10,120 A(BC) animals had both their line B maternal grandsire and their line C maternal granddam included in the data, and these were retained for further analyses. To investigate the sensitivity of results due to imposing a MAF threshold, all analyses were repeated using only the 51,237 SNPs that had a MAF higher than 0.1.

To position the broiler data relative to closely-related, distantly-related, and unrelated lines in the simulated data, we computed F_ST_ values [19] among the genotyped purebred animals for all datasets using the --fst option in PLINK [12] and report values averaged across all loci.

## Results

### Data

In the simulated data, F_ST_ values between lines were on average across replicates equal to 0.04, 0.12 and 0.22 for, respectively, closely-related, distantly-related, and unrelated lines. In the broiler data, the average F_ST_ value between the parental lines was 0.24, suggesting that the parental lines in the broiler data were further apart than the unrelated lines in the simulated data that separated 50 generations ago.

### Simulated data

The results for estimated line proportions for the simulated data using ADMIXTURE with various levels of LD pruning are in Table 1. Pruning the simulated data based on LD reduced the number of SNPs by 18–31% for an r^2^ threshold of 0.9, and by 95% for an r^2^ threshold of 0.1. For the same r^2^ threshold, fewer SNPs were removed if the lines were more closely related. For all scenarios, the accuracy was highest, and dispersion bias and RMSE were lowest, with pruning against an r^2^ threshold of 0.5. However, differences between scenarios were very small, apart from the scenario with an r^2^ threshold of 0.1, where results were considerably poorer. This is confirmed by correlations between estimated line proportions with different levels of pruning (see Additional file 1: Table S1). Those correlations are generally relatively close to 1 for scenarios with an r^2^ threshold of 0.3 or greater. With an r^2^ threshold of 0.1, the correlations with other levels of LD pruning were considerably lower, especially for the closely-related lines. Hereafter, the results obtained with an r^2^ threshold of 0.5 will be considered in the comparison of results between methods.

Comparison of estimated line proportions against the true values, showed that the accuracy of BOA was higher than 0.995 for the distantly-related and unrelated lines, and 0.986 for the closely-related lines (Table 2). Furthermore, the BOA results had little dispersion bias, very small RMSE, and relatively low maximum errors compared to the other methods. As a result, the observed distribution of estimated line proportions derived from BOA, followed the theoretically expected distribution closely (see Additional file 2: Fig. S1).

**Table 2:** Different quality measures of the estimated^a^ line B proportions when compared against true or BOA estimated values for the simulated data^b^

Measure	Reference	Lines	BOA	LR	ADM	REL_GP	REL_GP_noF
Accuracy	True	Close	0.986	0.680	0.692	0.949	0.954
		Distant	0.996	0.876	0.902	0.950	0.959
		Unrelated	0.997	0.950	0.967	0.952	0.962
	BOA	Close		0.690	0.704	0.936	0.942
		Distant		0.878	0.903	0.948	0.956
		Unrelated		0.948	0.965	0.951	0.961
Dispersion bias	True	Close	0.958	0.513	0.530	0.928	0.923
		Distant	0.964	0.776	0.820	0.966	0.958
		Unrelated	0.962	0.910	0.934	1.025	1.022
	BOA	Close		0.536	0.555	0.943	0.938
		Distant		0.804	0.849	0.996	0.987
		Unrelated		0.942	0.966	1.061	1.058
Maximum error	True	Close	0.076	0.321	0.317	0.109	0.105
		Distant	0.046	0.172	0.155	0.106	0.098
		Unrelated	0.038	0.109	0.089	0.104	0.094
	BOA	Close		0.312	0.308	0.124	0.120
		Distant		0.178	0.159	0.114	0.105
		Unrelated		0.114	0.089	0.109	0.099
RMSE	True	Close	0.019	0.109	0.106	0.036	0.035
		Distant	0.012	0.061	0.053	0.035	0.032
		Unrelated	0.010	0.037	0.030	0.035	0.032
	BOA	Close		0.108	0.104	0.041	0.039
		Distant		0.060	0.053	0.037	0.034
		Unrelated		0.038	0.031	0.037	0.033

*RMSE* root mean squared error

^a^Line B proportions are estimated from estimated breed-origin-of-alleles (BOA), using linear regression on mean allele counts within line (LR), ADMIXTURE analysis (ADM) after pruning SNPs based on r^2^ > 0.5, the genomic relationship with maternal grandsire (REL_GP), or this relationship after adjusting all self-relationships to be 1 (REL_GP_noF)

^b^Results are based on the average 188 A(BC) crossbreds with both maternal grandparents included with genotypes in the data

The LR method and ADMIXTURE gave similar results, with ADMIXTURE generally outperforming LR slightly. The LR method and ADMIXTURE were rather sensitive to the population structure, respectively achieving high accuracies of 0.950 and 0.967 with unrelated lines, but low accuracies of 0.680 and 0.692 with closely-related lines. In contrast, REL_GP and REL_GP_noF yielded respectively accuracies of  ~ 0.95 and  ~ 0.96 regardless of the population structure. For these four methods (LR, ADMIXTURE, REL_GP and REL_GP_noF), dispersion bias decreased when the relationships between the parental lines decreased, with little dispersion bias observed with unrelated lines. For the related lines REL_GP and REL_GP_noF yielded a regression coefficient of  ~ 0.92, but for LR and ADMIXTURE it dropped to almost 0.5, indicating very severe inflation of the variance of the estimated line proportions. Finally, the maximum error and the RMSE decreased considerably for LR and ADMIXTURE with decreasing relationships between the lines, while for REL_GP and REL_GP_noF these were not affected by the relationships between the lines, and in all cases were as low as for LR and ADMIXTURE for the unrelated lines.

Correlations between estimated line proportions using the various methods, showed that LR and ADMIXTURE gave very similar results, with the correlation increasing from 0.943 with closely-related lines to 0.975 with unrelated lines (Table 3). Correlations between REL_GP and REL_GP_noF were 0.989 or higher, showing that these methods yielded virtually the same results. Between the two groups of methods, i.e., LR and ADMIXTURE versus REL_GP and REL_GP_noF, correlations increased from  ~ 0.65 with closely-related lines to  ~ 0.91 with unrelated lines.

**Table 3:** Correlation among line B proportions estimated with different methods^a^ for the simulated data

Lines	Method	LR	ADM	REL_GP	REL_GP_noF
Closely-related	BOA	0.690	0.704	0.936	0.942
	LR	1	0.943	0.633	0.644
	ADM	0.943	1	0.647	0.659
	REL_GP	0.633	0.647	1	0.994
	REL_GP_noF	0.644	0.659	0.994	1
Distantly-related	BOA	0.878	0.903	0.948	0.956
	LR	1	0.963	0.817	0.834
	ADM	0.963	1	0.844	0.859
	REL_GP	0.817	0.844	1	0.991
	REL_GP_noF	0.834	0.859	0.991	1
Unrelated	BOA	0.948	0.965	0.951	0.961
	LR	1	0.975	0.890	0.905
	ADM	0.975	1	0.917	0.930
	REL_GP	0.890	0.917	1	0.989
	REL_GP_noF	0.905	0.930	0.989	1

^a^Line B proportions are estimated from estimated breed-origin-of-alleles (BOA), using linear regression on mean allele counts within line (LR), ADMIXTURE analysis (ADM), the genomic relationship with maternal grandsire (REL_GP), or this relationship after adjusting all self-relationships to 1 (REL_GP_noF)

The results obtained for LR, ADMIXTURE and both relationship-based approaches were very similar, when compared against BOA instead of the true line proportions (Table 2). The clearest difference was observed for the dispersion bias, where for all methods and scenarios the evaluation of the results against BOA resulted in a slight underestimation of the dispersion bias, with regression coefficients being 0.02–0.04 higher, and in nearly all cases closer to 1. These results, combined with the observed accuracy of the BOA estimated line proportions of almost 1, confirm that BOA can be used as a gold standard in empirical validation of estimated line proportions.

### Broiler data

**Fig. 1:**
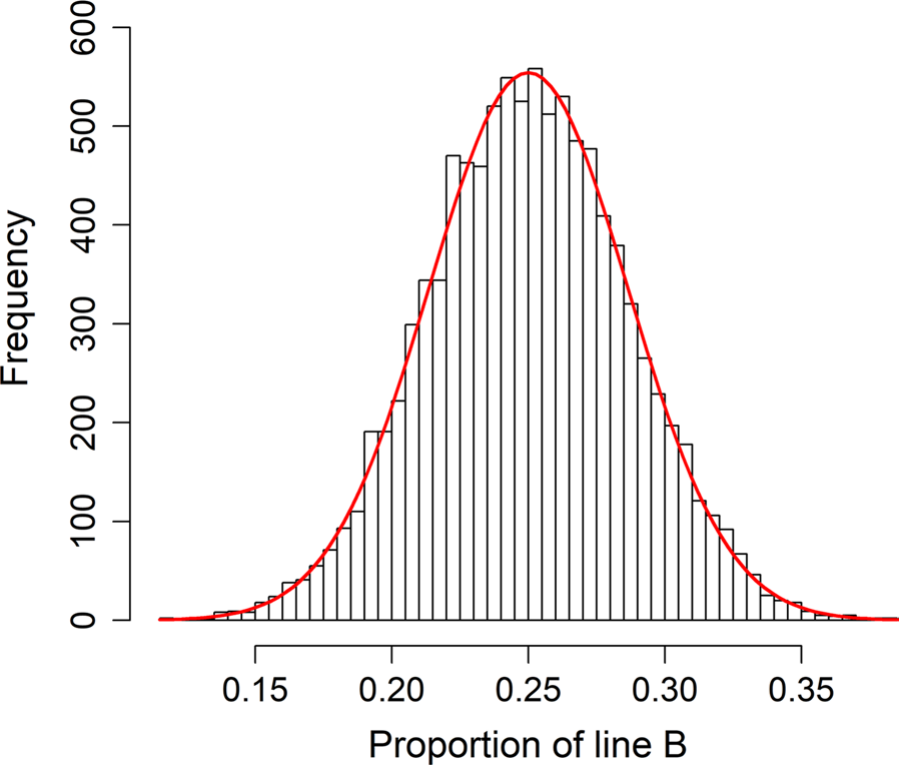
Observed line B proportions in the broiler data (histogram), versus the theoretically expected distribution (red line)

The observed distribution of estimated line B proportions derived from BOA followed the theoretically expected distribution very closely (Fig. 1). This suggests that the properties of the BOA-derived line composition are very similar to the properties of the true (unobserved) line composition.

The results for estimated line proportions for the broiler data using ADMIXTURE with various levels of LD pruning are in Table 4. Pruning the broiler data based on LD reduced the number of SNPs by 12% for an r^2^ threshold of 0.9, and by 94% for an r^2^ threshold of 0.1. Using BOA as a gold standard, similarly high accuracies and similarly low dispersion bias and RMSE were obtained after pruning against r^2^ thresholds of 0.5, 0.7 of 0.9. The results were only slightly poorer after pruning against an r^2^ threshold of 0.3 or when using all SNPs, albeit that the dispersion bias was somewhat lower in the latter case. Similar to the simulated data, the results after pruning against an r^2^ threshold of 0.1 were considerably worse than for any of the other thresholds. Hereafter, based on these results and to be consistent with the approach for the simulated data, the results obtained with an r^2^ threshold of 0.5 will be considered in the comparison of results across methods.

**Table 4:** Different quality measures of the estimated line B proportions derived with ADMIXTURE with various levels of pruning based on linkage disequilibrium (r^2^) in the broiler data

Measure	r^2^ threshold for pruning					
	0.1	0.3	0.5	0.7	0.9	No.
Number of SNPs	3392	15,144	29,892	41,376	48,905	55,729
Accuracy	0.839	0.914	0.927	0.927	0.925	0.918
Dispersion bias	0.970	1.140	1.138	1.128	1.115	1.090
Maximum error	0.094	0.073	0.066	0.064	0.068	0.070
RMSE	0.024	0.018	0.017	0.017	0.017	0.018

*RMSE* root mean squared error

Comparison of estimated line proportions of all four methods against BOA values, showed that the accuracy was highest with ADMIXTURE, closely followed by LR and REL_GP_noF (Table 5). The accuracy achieved with REL_GP was considerably lower. Dispersion bias appeared to be lowest with LR and highest with REL_GP_noF. The maximum error and RMSE were lowest and very similar with LR and ADMIXTURE, highest with REL_GP, and intermediate with REL_GP_noF. Removing SNPs with a MAF lower than 0.1 reduced the number of SNPs from 55,729 to 51,237, and hardly affected the results (see Additional file 1: Table S2).

**Table 5:** Different quality measures of the estimated^a^ line B and C proportions for the broiler data

Measure	LR	ADMIXTURE	REL_GP	REL_GP_noF
Accuracy	0.917	0.927	0.861	0.902
Dispersion bias	1.091	1.138	1.146	1.232
Max	0.068	0.066	0.149	0.102
RMSE	0.018	0.017	0.023	0.020

*RMSE* root mean squared error

^a^Line proportions are estimated using linear regression on mean allele counts within line (LR), ADMIXTURE analysis after pruning SNPs based on r^2^ > 0.5, the genomic relationship with maternal grandsire (REL_GP), or this relationship after adjusting all self-relationships to 1 (REL_GP_noF)

Correlations between estimated line proportions using the various methods (Table 6), showed that LR and ADMIXTURE also gave very similar results with the broiler data (correlation of 0.975), similarly as REL_GP and REL_GP_noF (correlation of 0.970). Between the two groups of methods, REL_GP had a correlation of  ~ 0.85 with LR and ADMIXTURE, while this correlation was  ~ 0.90 for REL_GP_noF.

**Table 6:** Correlation among line B proportions estimated with different methods^a^ for the broiler data

Method	LR	ADMIXTURE	REL_GP	REL_GP_noF
BOA	0.917	0.927	0.861	0.902
LR	1	0.975	0.843	0.890
ADMIXTURE	0.975	1	0.864	0.911
REL_GP	0.843	0.864	1	0.970
REL_GP_noF	0.890	0.911	0.970	1

^a^Line B proportions are estimated from estimated breed-origin-of-alleles (BOA), using linear regression on mean allele counts within line (LR), ADMIXTURE analysis, the genomic relationship with maternal grandsire (REL_GP), or this relationship after adjusting all self-relationships to 1 (REL_GP_noF)

## Discussion

Our objective was to compare the estimation of dam line composition in 3-way crossbred animals using different methods. In this section, we discuss the use of BOA-derived line composition as a gold standard, the performance of the different methods, and some implications for application in practice.

### Using BOA as a gold standard

We hypothesized that BOA is an appropriate gold standard for line composition, based on the fact that true line proportions are defined as the proportion of alleles derived from a particular line, and on our previous observation that assigning BOA at the allele level is highly accurate [7]. Indeed, the analysis of the simulated data showed that line proportions based on estimated BOA have an accuracy of almost 1, very limited estimation errors, while the variance of the estimated line proportions tends to be a little bit inflated. Coefficients of regressing BOA results instead of true values on the estimates from the other methods were generally slightly higher. This indicates that the actual bias of the scale of the estimates may be slightly greater than what the results using BOA as a gold standard suggest. Altogether, this shows that BOA-derived line composition is an appropriate gold standard in empirical comparisons of methods. Given that the same procedure as used here [7] was able to assign 43.5 to 45.7% of the alleles to the two dam lines in 3-way crossbred pigs [20], and 94.35% of all alleles in an F2 crossbred Girolando cattle population [21], it is expected that BOA can be used as a gold standard for other crosses in other species as well. Instead of the method that we developed and applied, BOA could be determined using other methods such as ChromoPainter [22].

### Performance of different methods

The well-established ADMIXTURE method yielded estimated line proportions that were competitive in terms of accuracy for the distantly-related lines, and were the most accurate for unrelated lines. For applications of ADMIXTURE, it is recommended to perform LD pruning, to try to meet the assumption that the markers are in linkage equilibrium [4]. Stringent LD pruning, i.e., against an r^2^ threshold of 0.1, resulted in the loss of  ~ 94% of the SNPs, and was detrimental to the accuracy of predicting line proportions. Therefore, pruning against an intermediate r^2^ threshold of e.g., 0.5 seems advisable, to avoid losing too many SNPs. The LR method yielded somewhat lower accuracies than ADMIXTURE, albeit that the correlations with estimates from ADMIXTURE were all higher than 0.94 (Tables 3 and 6). Coefficients of regression of true on estimated values showed that nearly all estimated line proportions had too much variance, i.e., regression coefficients were almost all less than 1. However, the obtained regression coefficients were closely related to the accuracies; lower accuracies were accompanied by lower regression coefficients. These results may suggest that the observed inflation of the variance of the estimates was due to post-processing of the estimates, of which the last step effectively proportionally scaled the proportions for both dam lines such that their sum was 0.5. Indeed, this scaling does increase the variance of the estimates while it is likely to introduce some error as well. Computing our results for the simulated data without post-processing (see Additional file 1: Table S3) confirmed that this is the case for the BOA-derived estimates. Before post-processing, the BOA-derived estimates showed virtually no dispersion bias, while the post-processing led to inflation of the variance of the estimates and a marginal increase of the accuracy. The post-processing reduced the inflation of the estimates for all other methods, and increased the accuracy for all other methods except LR. The likely explanation is that the post-processing step also involved forcing estimates within the interval of 0–0.5, which was never needed for the BOA results since they are within the interval of 0–0.5 by definition, while values outside this range were possible for all other methods. These changes are expected to improve the estimates, while reducing their variance. The observation that the post-processing step hardly affected the accuracy of LR, may be due to the fact that with LR the expected contribution of the sire line is first removed from the genotypes, which reduces the errors that can be made. As a result, the post-processing step does not yield the improvement that is observed for the other methods.

Comparisons of methods to predict line composition have been done previously, considering at least partly different methods. The ADMIXTURE method uses the same likelihood model as implemented in STRUCTURE [23], and therefore both methods typically yield similar estimates [4]. Frkonja et al. [9] showed that partial least squares regression, BayesB [24] and LASSO [25], which are essentially all linear variable selection methods, yielded very similar breed composition estimates using 50k SNPs in the admixed Swiss Fleckvieh as STRUCTURE. Dodds et al. [26] showed that estimates of breed composition in New Zealand sheep based on 50k genotypes using linear regression or genomic best linear unbiased prediction (GBLUP), also a linear model, yielded similar estimates to those obtained with STRUCTURE. These reported results confirm our finding that estimates of breed composition based on 50k genotypes and using linear models, typically give very similar results to those obtained with ADMIXTURE and STRUCTURE.

For LR and ADMIXTURE, the accuracy increased steeply with increasing distance between the lines, while this effect was much less pronounced with the REL_GP methods. In fact, for the closely-related lines in the simulated data, the REL_GP methods achieved acceptable accuracies (> 0.95), while this was not the case for ADMIXTURE and LR (< 0.7). Similar results were observed in a study that estimated the breed composition of Brangus, a composite breed of Brahman and Angus, and Beefmaster cattle, a composite breed assumed to be about 25% Hereford, 25% Milking Shorthorn, and 50% Brahman [27]. That study compared estimates of LR and ADMIXTURE to genomic breed compositions that were computed from path analysis either considering only the relationships to the ancestral breeds directly (termed D-GBC), or additionally considering the genomic similarities between the ancestral breeds (termed C-GBC). All methods showed similar results for the Brangus breed, whose ancestral breeds are distantly related. For the Beefmaster breed, however, the high genomic similarities between Hereford and Shorthorn impaired the performance of LR, ADMIXTURE and C-GBC, while the performance of D-GBC was much more robust against the ancestral breeds being closely related [27]. Given that the assumptions underlying C-GBC are effectively closer to those of LR, while D-GBC models the direct inheritance to the ancestral breed similarly to the REL_GP and BOA methods, our results are very much in line with those of Wu et al. [27].

The main benefits of the LR method are that it is computationally efficient, and easy to implement. This makes it relatively straightforward to implement accounting for the known contribution of the sire line before applying the LR method for the dam lines. Arguably, this step is merely pre-processing of the data. A more sophisticated approach would be to remove the actual haplotype contributed by the sire, instead of the expected contribution of an average sire. This may further improve the accuracy. However, this does require that phased genotypes of the sires are available and it would increase the computational burden. The post-processing steps used to ensure that dam lines contributions were 0.25 on average and summed to 0.5 within animal, could more formally be integrated in the LR method. This has been done previously by using constrained regression that ensured that all estimates are within the parameter space, i.e., estimated line proportions are within the 0–1 interval, while the sum of the estimated line proportions is constrained to be 1 [8]. This constrained linear regression yielded accurate estimates of breed composition in an admixed population, when considering all 11 founder breeds in the model, while the accuracy of ADMIXTURE was considerably lower in their study. When considering only one founder breed in the model and an average of allele frequencies for the remaining breeds, the constrained linear regression and ADMIXTURE yielded very similar results [8], in line with our results. Similarly, a Bayesian method has been proposed that guarantees estimates to be within the parameter space, which showed higher accuracy in a multibreed Angus-Brahman population compared to linear regression, while estimates between both methods had a high correlation of  ~ 0.92 [10].

Both our REL_GP methods gave highly accurate estimates, and this was hardly affected by the distance between the lines. Based on this, especially for closely-related lines, REL_GP_noF is the best method. Implementation in practice is relatively straightforward, albeit that it requires that the genotypes of all grandparents are available. In addition, applying this method in more complex crosses may be more tedious for animals that have contributions of a particular breed both from their sire and dam. Based on the results from the simulated data, it is advisable to first compute the F_ST_ between the parental lines, to inform the choice of method to use. Based on the observation that our broiler data were comparable to the unrelated lines in the simulated data, while previously pig data were shown to be comparable to the distantly-related lines [7], it can be concluded that for many applications in pigs and poultry the LR method is a very competitive method to derive dam line composition. In other applications where the involved breeds or lines are closely related, more sophisticated models are needed that somehow consider the inheritance from each ancestral breed more closely. This can be achieved by tracing the inheritance of long-range haplotypes such as done with BOA, by using the relationship to the purebred ancestor of the corresponding breed as in the REL_GP methods, or by decomposing the relationships to the purebred ancestors using e.g., path analysis [27].

### Implications—applications in animal breeding

In our study, we assumed that 50k genotypes would be available for all animals. For most breeding programs, indeed either all animals are genotyped for 50k genotypes, or certain groups of animals are genotyped at lower density and then imputed up to 50k before being used in genomic breeding value estimation. Nevertheless, there may be situations where imputation is cumbersome or inaccurate, such that it may be preferable to derive the line composition based on a smaller subset of SNPs for which all the animals are actually genotyped. Kuehn et al. [6] showed that using the Illumina Bovine3K instead of the BovineSNP50, reduced the concordance with pedigree-based breed composition from 89 to 83%. Frkonja et al. [9] showed that the estimated admixture in Swiss Fleckvieh cattle using 4000 equally-spaced SNPs was very similar to that using all 50k SNPs, and that selecting SNPs based on F_ST_ values could yield very similar estimates with as few as  ~ 500 SNPs. Previously, it was suggested that if a targeted small SNP panel is used to derive breed composition, including low MAF SNPs, it may help to obtain more accurate predictions [28]. Our results from the broiler data showed that removing SNPs with a MAF lower than 0.1 in the entire data hardly affects the results, suggesting that applying the usual low MAF filtering in 50k SNPs does not affect the estimated line or breed composition.

Our implementation of the LR method was developed specifically for the dam lines of a 3-way cross, as well as the post-processing of results of all methods to meet expectations of the line composition both within and across 3-way crossbred animals, implying that we assumed that all the 3-way crossbred animals indeed belonged to this breed category. However, the methods presented could also be applied to verify or establish the type of cross in the first place, for instance to check if none of the animals actually were F1’s rather than 3-way crossbred animals. In such applications, the rules that we applied in the LR method should be omitted, as well as the post-processing step applied for all methods. Thus, estimation of line composition of crossbred animals may have to be done twice: the first time to confirm the type of cross, considering all possible lines involved in the crossbred animal, and the second time to refine estimated line proportions using the then established or confirmed type of cross. These estimated line proportions could then be used in breeding value estimation to model the contribution of both dam lines to the crossbred animals, rather than considering the same effect for all animals belonging to the same cross. Whether or not such refined modelling of line composition affects estimated genomic breeding values, likely depends on the differences in genetic level between the dam lines for the various breeding goal traits.

## Conclusions

The dam line contributions in 3-way crossbred animals can be very accurately estimated as the proportions of alleles that are assigned to the different dam lines, based on comparing phased genotypes of crossbred animals against haplotype libraries of the purebred parental lines that are involved in the crossbreeding program. Therefore, these BOA-derived dam line proportions can be used as a gold standard to empirically validate methods to estimate the dam line proportions that are much easier to implement, and computationally less demanding. Of all considered methods in this study, the relationships with the maternal grandparents achieved the highest accuracy, and were only marginally affected if the maternal lines were more closely related to each other. We showed that linear regression of the crossbred genotypes on line allele frequencies and ADMIXTURE achieved similar accuracy for unrelated parental lines as the relationship with the maternal grandparent, but much lower accuracy if the parental lines were separated 20 or less generations ago. Nevertheless, parental lines in most pig and poultry crosses are likely more distantly related than that, suggesting that ADMIXTURE and LR are appropriate methods to predict dam line contribution in 3-way crossbred animals. Moreover, LR is straightforward to implement and can be easily adapted to consider the specific nature of the crossbred animals analysed. Finally, for almost all the methods, there was some benefit from adjusting estimates to fit within the parameter space, i.e., by ensuring that the sum of dam line contributions within animals was equal to 0.5, and that within dam line and across animals the average was equal to 0.25.

## Supplementary Information

**Additional file 1: Table S1.** Correlation among line B proportions estimated with ADMIXTURE with different levels of pruning for the simulated data. **Table S2.** Different quality measures of the estimated line B and C proportions for the broiler data based on 51,237 SNPs with a minor allele frequency  > 0.1. **Table S3.** Different quality measures of the estimated line B proportions without any post-processing when compared against true values for the simulated data.

**Additional file 2: Figure S1.** Observed line B proportions in the simulated data (histogram), versus the theoretically expected distribution (red line).

## Data Availability

The simulated data is available via https://doi.org/10.4121/17198453. The broiler genotype data used in the present study were provided by Cobb-Vantress, Inc. and are not publicly accessible, but only available through Cobb-Vantress.

## References

[1] Duenk P, Bijma P, Wientjes YCJ, Calus MPL (2021). Review: optimizing genomic selection for crossbred performance by model improvement and data collection. J Anim Sci.

[2] Xiang T, Christensen OF, Vitezica ZG, Legarra A (2016). Genomic evaluation by including dominance effects and inbreeding depression for purebred and crossbred performance with an application in pigs. Genet Sel Evol.

[3] Sevillano CA, Vandenplas J, Bastiaansen JWM, Bergsma R, Calus MPL (2017). Genomic evaluation for a three-way crossbreeding system considering breed-of-origin of alleles. Genet Sel Evol.

[4] Alexander DH, Novembre J, Lange K (2009). Fast model-based estimation of ancestry in unrelated individuals. Genome Res.

[5] Chiang CWK, Gajdos ZKZ, Korn JM, Kuruvilla FG, Butler JL, Hackett R (2010). Rapid assessment of genetic ancestry in populations of unknown origin by genome-wide genotyping of pooled samples. PLoS Genet.

[6] Kuehn LA, Keele JW, Bennett GL, McDaneld TG, Smith TPL, Snelling WM (2011). Predicting breed composition using breed frequencies of 50,000 markers from the US Meat Animal Research Center 2,000 Bull Project. J Anim Sci.

[7] Vandenplas J, Calus MPL, Sevillano CA, Windig JJ, Bastiaansen JWM (2016). Assigning breed origin to alleles in crossbred animals. Genet Sel Evol.

[8] Boerner V, Wittenburg D (2018). On estimation of genome composition in genetically admixed individuals using constrained genomic regression. Front Genet.

[9] Frkonja A, Gredler B, Schnyder U, Curik I, Sölkner J (2012). Prediction of breed composition in an admixed cattle population. Anim Genet.

[10] Martínez CA, Khare K, Elzo MA (2018). BIBI: Bayesian inference of breed composition. J Anim Breed Genet.

[11] Alexander DH, Lange K (2011). Enhancements to the ADMIXTURE algorithm for individual ancestry estimation. BMC Bioinformatics.

[12] Chang CC, Chow CC, Tellier LCAM, Vattikuti S, Purcell SM, Lee JJ (2015). Second-generation PLINK: rising to the challenge of larger and richer datasets. GigaScience.

[13] Wientjes YCJ, Bijma P, Vandenplas J, Calus MPL (2017). Multi-population genomic relationships for estimating current genetic variances within and genetic correlations between populations. Genetics.

[14] He J, Guo Y, Xu J, Li H, Fuller A, Tait RG (2018). Comparing SNP panels and statistical methods for estimating genomic breed composition of individual animals in ten cattle breeds. BMC Genet.

[15] Hill WG, Weir BS (2011). Variation in actual relationship as a consequence of Mendelian sampling and linkage. Genet Res.

[16] Groenen MAM, Wahlberg P, Foglio M, Cheng HH, Megens HJ, Crooijmans RPMA (2009). A high-density SNP-based linkage map of the chicken genome reveals sequence features correlated with recombination rate. Genome Res.

[17] Ramos AM, Crooijmans RPMA, Affara NA, Amaral AJ, Archibald AL, Beever JE (2009). Design of a high density SNP genotyping assay in the pig using SNPs identified and characterized by next generation sequencing technology. PLoS One.

[18] Calus MPL, Vandenplas J, Hulsegge I, Borg R, Henshall JM, Hawken R (2019). Assessment of sire contribution and breed-of-origin of alleles in a three-way crossbred broiler dataset. Poult Sci.

[19] Weir BS, Cockerham CC (1984). Estimating F-statistics for the analysis of population structure. Evolution.

[20] Sevillano CA, Vandenplas J, Bastiaansen JWM, Calus MPL (2016). Empirical determination of breed-of-origin of alleles in three-breed cross pigs. Genet Sel Evol.

[21] Otto PI, Guimarães SEF, Calus MPL, Vandenplas J, Machado MA, Panetto JCC (2020). Single-step genome-wide association studies (GWAS) and post-GWAS analyses to identify genomic regions and candidate genes for milk yield in Brazilian Girolando cattle. J Dairy Sci.

[22] Lawson DJ, Hellenthal G, Myers S, Falush D (2012). Inference of population structure using dense haplotype data. PLoS Genet.

[23] Pritchard JK, Stephens M, Donnelly P (2000). Inference of population structure using multilocus genotype data. Genetics.

[24] Meuwissen THE, Hayes BJ, Goddard ME (2001). Prediction of total genetic value using genome-wide dense marker maps. Genetics.

[25] Tibshirani R (1996). Regression shrinkage and selection via the Lasso. J R Stat Soc Series B Methodol.

[26] Dodds KG, Auvray B, Newman SA, McEwan JC (2014). Genomic breed prediction in New Zealand sheep. BMC Genet.

[27] Wu XL, Li Z, Wang Y, He J, Rosa GJM, Ferretti R (2020). A causality perspective of genomic breed composition for composite animals. Front Genet.

[28] Pant SD, Schenkel FS, Verschoor CP, Karrow NA (2012). Use of breed-specific single nucleotide polymorphisms to discriminate between Holstein and Jersey dairy cattle breeds. Anim Biotechnol.

