# Supplementary material for: Estimation of dam line composition of 3-way crossbred animals using genomic information
Source: Genet Sel Evol. 2022 Jun 15;54:44. doi: 10.1186/s12711-022-00728-4 (PMC9199202; doi:10.1186/s12711-022-00728-4)
Supplement: Supplementary file 1 — Additional file 1: Table S1. Correlation among line B proportions estimated with ADMIXTURE with different levels of pruning for the simulated data. Table S2. Different quality measures of the estimated line B and C proportions for the broiler data based on 51,237 SNPs with a minor allele frequency > 0.1. Table S3. Different quality measures of the estimated line B proportions without any post-processing when compared against true values for the simulated data. [file 12711_2022_728_MOESM1_ESM.docx]

**Additional file 1 Table S1**

**Table S1 Correlation among line B proportions estimated with ADMIXTURE with different levels of pruning for the simulated data**

| Lines | r^2^ pruning | 0.1 | 0.3 | 0.5 | 0.7 | 0.9 | No |
| --- | --- | --- | --- | --- | --- | --- | --- |
| Closely-related | 0.1 | 1 | 0.790 | 0.787 | 0.788 | 0.787 | 0.782 |
|  | 0.3 | 0.790 | 1 | 0.957 | 0.947 | 0.938 | 0.927 |
|  | 0.5 | 0.787 | 0.957 | 1 | 0.987 | 0.979 | 0.970 |
|  | 0.7 | 0.788 | 0.947 | 0.987 | 1 | 0.993 | 0.985 |
|  | 0.9 | 0.787 | 0.938 | 0.979 | 0.993 | 1 | 0.993 |
|  | No | 0.782 | 0.927 | 0.970 | 0.985 | 0.993 | 1 |
| Distantly-related | 0.1 | 1 | 0.877 | 0.876 | 0.877 | 0.876 | 0.872 |
|  | 0.3 | 0.877 | 1 | 0.973 | 0.967 | 0.960 | 0.955 |
|  | 0.5 | 0.876 | 0.973 | 1 | 0.991 | 0.984 | 0.979 |
|  | 0.7 | 0.877 | 0.967 | 0.991 | 1 | 0.996 | 0.991 |
|  | 0.9 | 0.876 | 0.960 | 0.984 | 0.996 | 1 | 0.996 |
|  | No | 0.872 | 0.955 | 0.979 | 0.991 | 0.996 | 1 |
| Unrelated | 0.1 | 1 | 0.918 | 0.919 | 0.922 | 0.922 | 0.919 |
|  | 0.3 | 0.918 | 1 | 0.980 | 0.976 | 0.971 | 0.966 |
|  | 0.5 | 0.919 | 0.980 | 1 | 0.993 | 0.988 | 0.983 |
|  | 0.7 | 0.922 | 0.976 | 0.993 | 1 | 0.997 | 0.992 |
|  | 0.9 | 0.922 | 0.971 | 0.988 | 0.997 | 1 | 0.997 |
|  | No | 0.919 | 0.966 | 0.983 | 0.992 | 0.997 | 1 |

**Additional file 1 Table S2**

**Table S2 Different quality measures of the estimated^a^ line B and C proportions for the broiler data based on 51,237 SNPs with a minor allele frequency > 0.1**

| Measure | LR | ADMIXTURE | REL_GP | REL_GP_noF |
| --- | --- | --- | --- | --- |
| Accuracy | 0.916 | 0.915 | 0.861 | 0.901 |
| Dispersion bias | 1.086 | 1.077 | 1.189 | 1.279 |
| Max | 0.068 | 0.069 | 0.148 | 0.101 |
| RMSE | 0.018 | 0.017 | 0.023 | 0.021 |

^a^Line proportions are estimated using linear regression on mean allele counts within line (LR), ADMIXTURE analysis after pruning SNPs based on r^2^ > 0.5, the genomic relationship with maternal grandsire (REL_GP), or this relationship after adjusting all self-relationships to 1 (REL_GP_noF).

RMSE: root mean squared error

**Additional file 1 Table S3**

**Table S3 Different quality measures of the estimated^a^ line B proportions without any post-processing when compared against true values for the simulated data**

| Measure | Lines | BOA | LR | ADM | REL_GP | REL_GP_noF |
| --- | --- | --- | --- | --- | --- | --- |
| Accuracy | Close | 0.981 | 0.682 | 0.629 | 0.912 | 0.931 |
|  | Distant | 0.994 | 0.875 | 0.863 | 0.902 | 0.931 |
|  | Unrelated | 0.996 | 0.949 | 0.956 | 0.909 | 0.938 |
| Dispersion | Close | 1.009 | 0.453 | 0.420 | 0.855 | 0.868 |
| bias | Distant | 0.998 | 0.749 | 0.757 | 0.903 | 0.914 |
|  | Unrelated | 0.993 | 0.898 | 0.908 | 0.939 | 0.970 |
| Maximum | Close | 0.099 | 0.368 | 0.382 | 0.157 | 0.134 |
| error | Distant | 0.057 | 0.182 | 0.187 | 0.160 | 0.119 |
|  | Unrelated | 0.047 | 0.113 | 0.100 | 0.166 | 0.136 |
| RMSE | Close | 0.027 | 0.125 | 0.131 | 0.050 | 0.044 |
|  | Distant | 0.016 | 0.063 | 0.064 | 0.053 | 0.043 |
|  | Unrelated | 0.014 | 0.037 | 0.035 | 0.058 | 0.048 |

^a^Line B proportions are estimated from estimated breed-origin-of-alleles (BOA), using linear regression on mean allele counts within line (LR), ADMIXTURE analysis (ADM) after pruning SNPs based on r^2^ > 0.5, the genomic relationship with maternal grandsire (REL_GP), or this relationship after adjusting all self-relationships to 1 (REL_GP_noF).

RMSE: root mean squared error
