# Supplementary material for: Estimation of dam line composition of 3-way crossbred animals using genomic information
Source: Genet Sel Evol. 2022 Jun 15;54:44. doi: 10.1186/s12711-022-00728-4 (PMC9199202; doi:10.1186/s12711-022-00728-4)
Supplement: Supplementary file 2 — Additional file 2: Figure S1. Observed line B proportions in the simulated data (histogram), versus the theoretically expected distribution (red line). [file 12711_2022_728_MOESM2_ESM.docx]

**Additional file 2 Figure S1**

**Figure S1 Observed line B proportions in the simulated data (histogram), versus the theoretically expected distribution (red line)**

**
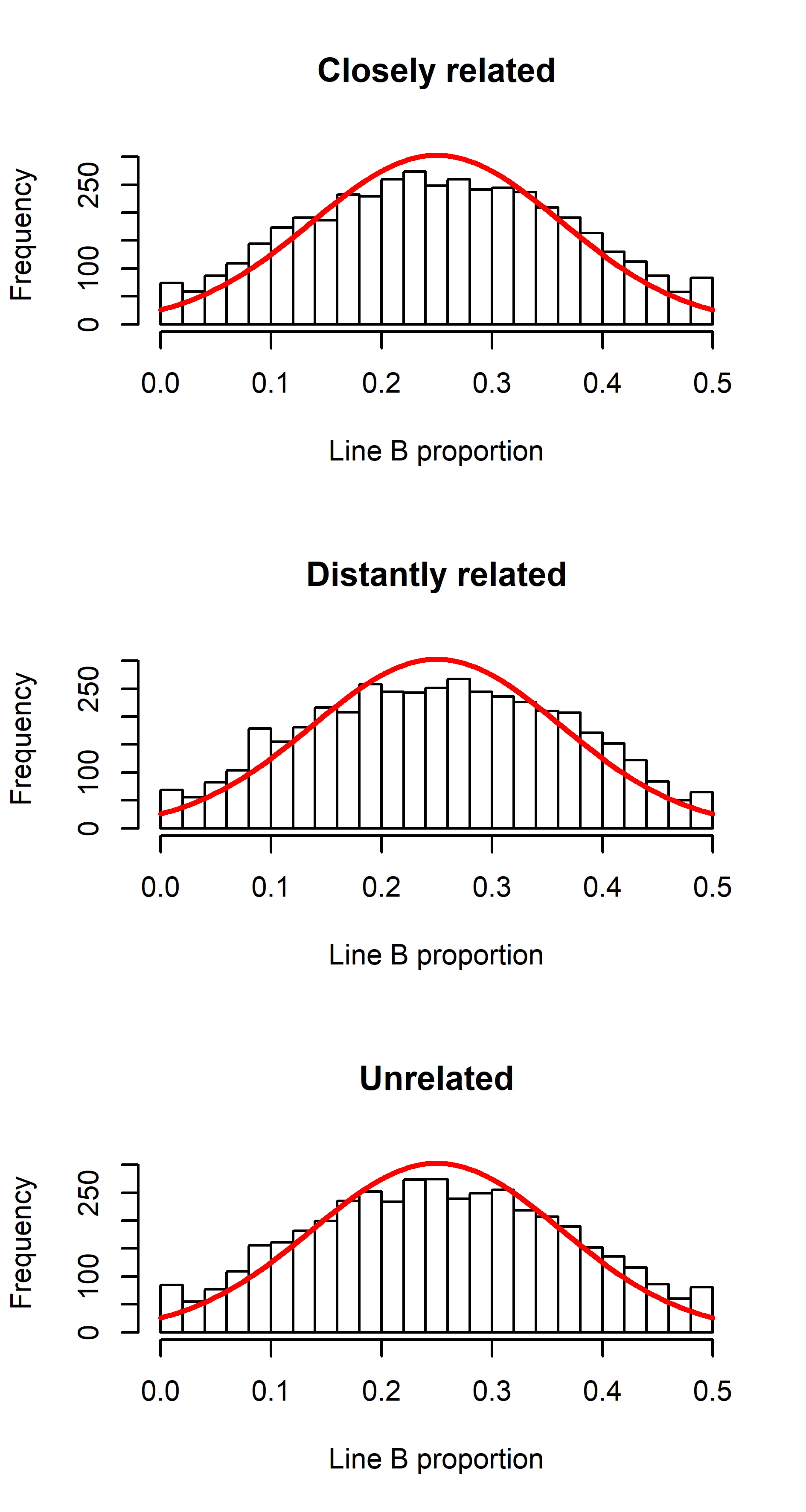
**
